# Supplementary material for: WordCommentsAnalyzer: A windows software tool for qualitative research
Source: F1000Res. 2018 Sep 4;7:536. Originally published 2018 May 3. [Version 2] doi: 10.12688/f1000research.14819.2 (PMC6137411; doi:10.12688/f1000research.14819.2)
Supplement: Supplementary file 3 [file f1000research-7-17614-s0002.tgz › b2a79496-4ada-47c4-9682-a99ce867836d.zip!/Eight Iranian Health Journals/The Journal of Toloo-e-Behdasht_4.docx]

© The Journal of Toloo-e-Behdasht. This work is licensed under CC BY 4.0 (https://creativecommons.org/licenses/by/4.0/).

Volume & Issue: دوره 16 - شماره 4

Title: مطالعه و پهنه بندی آلودگی شهر یزد به فلزات سنگین با استفاده از پر پرندگان به عنوان شاخص زیستی محیط زیست شهری

http://tbj.ssu.ac.ir/article-1-2250-fa.html

مقدمه: استفاده از شاخص‌های زیستی مانند پر پرندگان می‌تواند تصویر بهتری از انتقال‌زیستی آلاینده‌ها در اجزای محیط‌زیست را نشان دهد. مطالعه حاضر باهدف بررسی و پهنه‌بندی آلودگی فلزات سنگین در شاخص‌زیستی پر قمری‌خانگی انجام گرفت. روش بررسی: فلزات سنگین سرب و کادمیوم در دو زون غربی و شرقی شهر یزد با استفاده از پرقمری‌خانگی (Streptopelia senegalensis) به عنوان شاخص آلودگی محیط‌زیست مطالعه شد. جهت اندازه‌گیری غلظت فلزات سنگین از روش هضم شیمیایی و دستگاه جذب اتمی استفاده گردید. در آخر نتایج به دست آمده با استفاده از روش کریجینگ در محیط GIS پهنه‌بندی شد. یافته‌ها: در بررسی‌ نمونه‌های مورد مطالعه میانگین غلظت  سرب µg g-1 01/1 ± 88/19 و برای کادمیوم µg g-1 03/0 ± 87/0 به دست آمد. غلظت فلز سرب حداقل  µg g-187/13 و حداکثر µg g-1 35/31 و برای فلز کادمیوم حداقل  µg g-1 42/0 و حداکثر µg g-1 29/1 به دست آمد. فلز سرب دارای غلظتی بالاتر و فلز کادمیوم دارای غلظتی پایین‌تر از حد آستانه‌ی خطر برای پرندگان بود. نقشه‌های پهنه‌بندی در GIS توزیع مقدار آلودگی بالاتر در نیمه‌ی شرقی شهر یزد را نشان داد. نتایج پهنه‌بندی و آنالیزهای آماری غلظت فلزات‌سنگین سرب و کادمیوم در شهر یزد مشابه یکدیگر و حاکی از آلایندگی بالاتر نیمه شرقی شهر یزد بوده‌است. نتیجه‌گیری: الگوی آلودگی شهر یزد نشان دهنده تأثیرات فعالیت‌های انسانی و انتقالات محیطی آلاینده‌ها بر افزایش انتشار فلزات سنگین در نیمه‌ی شرقی شهر یزد بوده است. مطالعه حاضر نشان داد قمری‌خانگی دارای ویژگی‌های زیستی مناسب برای استفاده به عنوان شاخص زیستی آلودگی محیط‌زیست شهری بوده‌است.

Title: بررسی نیازهای آموزش بهداشت کارگران شرکت سنگ آهن مرکزی ایران – بافق در سال 1393

http://tbj.ssu.ac.ir/article-1-1581-fa.html

مقدمه :عصرما عصر برنامه ریزی است برنامه ریزی به خصوص در مورد نیازهای آموزشی مردم از اهمیت خاصی برخورداراست هدف این مطالعه  بررسی نیازهای آموزشی کارگران شرکت سنگ آهن مرکزی بافق  و اولویت بندی نیازها و تدوین طرح درس آموزشی براساس اولویتها  می باشد روش بررسی: این پژوهش یک مطالعه کیفی با استفاده ازتکنیک FGD (بحث گروهی) و با حضور 12 نفر از متخصصین حوزه سلامت و 46 نفر ازکارگران شرکت سنگ آهن صورت گرفت داده های به دست آمده براساس روش تحلیل محتوامورد تجزیه و تحلیل قرارگرفت. پس از جمع آوری پاسخ ها با استفاده از جدول ماتریکس  و گروه اسمی ،نیازها اولیت بندی گردید . یافته ها: نیازها و مشکلات احساس شده توسط  متخصصین و کارگران با یکدیگر متفاوت بود در کل کد های مفهومی 93 کد در 5درونمایه اصلی قرار گرفت از نظر متخصصین بهداشتی ؛ بیشترین مسئله موردنیاز کارگران بعد بیماریهای شغلی در حالیکه از نظر کارگران ،سلامت روان بود. نتیجه گیری: به نظر می رسد متخصصین به دلیل توجه به جنبه جسمانی سلامت ، بعد روانی را کمتر مد نظر قراردادند وبرنامه آموزشی موجود، برآورنده نیازهای آموزشی کارگران در ارتباط با مسائل بهداشتی نیست.بنابراین بایستی محتوای آموزشی متناسب با نیازهای بهداشتی کارگران طراحی و اجرا شود.

Title: بررسی فرهنگ ایمنی وعوامل موثر برآن دریک صنعت فولاد

http://tbj.ssu.ac.ir/article-1-2305-fa.html

مقدمه: فرهنگ ایمنی بیان کننده ارزش‌ها، نگرش‌ها، ادراکات، تلاش‌ها و الگوهای رفتاری فردی یا  گروهی است که تعهد، موفقیت و کارایی یک سیستم مدیریت ایمنی را تعیین می‌کند.هدف ازانجام مطالعه حاضر بررسی فرهنگ ایمنی وعوامل موثربرآن دریکی ازصنایع فولادمی باشد. روش بررسی: آشناترین ابزار برای اندازه گیری جنبه های روانشناختی ، پرسشنامه فرهنگ ایمنی است. در این مطالعه توصیفی – تحلیلی با استفاده ازپرسشنامه استاندارد، 202 نفر از پرسنل یک شرکت تولیدکننده فولاد مورد بررسی قرارگرفتند .این پرسشنامه دارای 74 سوال در دو بخش اطلاعات جامعه شناختی و سوالات سنجش فرهنگ ایمنی می باشد . داده های جمع‌آوری شده به نرم‌افزار spss (نسخه19)منتقل  و با استفاده از آمار توصیفی و تحلیلی ،نتایج تحلیل گردید. یافته ها:میانگین نمره نهایی فرهنگ ایمنی درافراد شرکت کننده دراین تحقیق 07/22±98/167 از نمره کلی 325 می باشد. اختلاف نمره فرهنگ ایمنی درگروههای مختلف تحصیلی به لحاظ آماری معنی داراست(02/0P =). علاوه بر این، اختلاف نمره کلی فرهنگ ایمنی در افراد حادثه دیده و حادثه ندیده از لحاظ آماری معنی دارمی باشد(02/0P =). نتیجه گیری: با توجه به یافته ها،فرهنگ ایمنی بیشترین همبستگی را با بعد تعهد مدیریت دارد. مدیریت سازمان با حمایت در طرح‌ریزی و اجرای برنامه های مناسب ایمنی نقش مهمی در ایجاد، ترویج و ارتقاء فرهنگ ایمنی داردکه منجربه ایمن ترشدن رفتارها ،افزایش فرهنگ ایمنی ودرنتیجه کاهش آمارحوادث سازمان می گردد.

Title: ترجمه، تعیین روایی و پایایی گونه فارسی ابزار استاندارد IWQOL-lite

http://tbj.ssu.ac.ir/article-1-1916-fa.html

مقدمه:چاقی یک بیماری مزمن با شیوه بالا و پنجمین عامل مرگ در جهان است که روند آن در حال افزایش می‌باشد. برای بررسی اثر درمان و شیوه های درمانی از پرسشنامه های کیفیت زندگی استفاده می‌شود. بنابراین هدف از مطالعه حاضر ترجمه، ‌پایایی و روایی پرسشنامه اختصاصی کیفیت زندگی چاقی در ایران می باشد. روش بررسی:مطالعه حاضر به روش توصیفی- تحلیلی بر روی 306 نفر از افراد بالای 18 سال ساکن در شهر یزد با روش نمونه گیری خوشه ای انجام گرفت. آزمون های پایایی مطالعه با استفاده از روش همبستگی درونی و ضریب توافق و روایی آن با استفاده از روایی همزمان، افتراقی و سازه با استفاده از نرم افزارها 16SPSS  و 6Mplus  انجام گرفت. یافته ها:میانگین سنی پاسخ دهندگان 97/33 سال بوده است.  123 نفر زن و183 نفر مرد بوده اند و اکثریت نمونه را افراد متاهل(6/78 درصد ) تشکیل داده اند.  نتایج حاصل از همخوانی درونی بیانگر این بود که در تمامی مقیاس ها گونه فارسی پرسشنامه IWQOL-lite از حداقل ضریب همبستگی پایایی  (97/0-84/0)برخوردار بوده است. ضریب توافق درونی که به منظور بررسی مناسب بودن آزمون مجدد انجام شد، در تمام مقیاس ها به جز عملکرد جنسی(57/0) مناسب (بیشتر از 70/0) بود. روایی افتراقی بیانگر این بود که این پرسشنامه قادر به افتراق زیر گروه های جنسی و وزنی است. شاخص های برازش مدل تحلیل عاملی تاییدی نیز بیانگر مناسب بودن روایی سازه بوده است. نتیجه گیری: گونه فارسی  پرسشنامهIWQOL-lite به منظور بررسی کیفیت زندگی افراد چاق و اضافه وزن از روایی و پایایی مناسب در جامعه ایرانی  برخوردار بوده است و توسط پزشکان و متخصصان تغذیه می تواند مورد اسفاده قرار بگیرد.

Title: بررسی میزان شیوع و مقاومت آنتی بیوتیکی لیستریا مونوسیتوژنز درمراحل مختلف کشتار مرغ و محیط کشتارگاه مرغ شهرستان یزد در سال ۱۳۹۴

http://tbj.ssu.ac.ir/article-1-2264-fa.html

مقدمه: لیستریا مونوسیتوژنز باکتری بیماری­زایی است که توسط مواد غذایی از جمله گوشت مرغ منتقل می شود و عامل بیماری لیستریوز می باشد. علایم شدیدی مانند مننژیت، سپتی سمی و سقط جنین ایجاد می کند. با توجه به اطلاعات اندک از آلودگی گوشت مرغ به لیستریا مونوسیتوژنز در شهرستان یزد و بروز مقاومت آنتی بیوتیکی در باکتری ها، هدف این مطالعه، تعیین شیوع و مقاومت آنتی بیوتیکی لیستریا مونوسیتوژنز جدا شده در مراحل مختلف کشتار مرغ و محیط کشتارگاه مرغ شهرستان یزد بود. روش بررسی: برای تعیین حضور لیستریا مونوسیتوژنز از محیط های کشت و آزمون های بیوشیمیایی استفاده شد، سپس با استفاده از روش PCR تایید شد و در نهایت تست آنتی بیوگرام برای تعیین مقاومت آنتی­بیوتیکی انجام پذیرفت. یافته ها: از مجموع 811 نمونه اخذ شده از دو کشتارگاه طیور یزد 104 نمونه (8/12 درصد) آلوده به لیستریا مونوسیتوژنزبودند. بیشترین آلودگی به سطوح پیش سرد و کمترین آلودگی مربوط به لاشه مرغ قبل از چیلر بود و بیشترین مقاومت آنتی بیوتیکی را نسبت به آمپی سیلین، تتراساکلین و پنی سیلین داشتند. نتیجه گیری: نتایج این بررسی حاکی از آلودگی گوشت مرغ و مقاومت آنتی بوتیکی لیستریا مونوسیتوژنز جدا سازی شده می باشد. با توجه به مصرف بالای گوشت مرغ و حمل ونقل آن به صورت خام تا محل عرضه، نبود کنترل مناسب و از طرفی استفاده از آنتی بیوتیک در طول پرورش می تواند سبب بروز خطرات جدی در سلامت جامعه شود. عدم آگاهی در مورد لیستریوز، ضرورت اجرای برنامه های آموزشی و اطلاع رسانی در زمینه ی ایمنی مواد غذایی و حتی پرورش را آشکار و ضرورت تدوین و اجباری شدن استاندارد جستجوی لیستریا در مواد غذایی حساس، لازم به نظر می رسد.

Title: بررسی میزان سرانه مصرف آب خانگی و عوامل مرتبط با آن در شهر اردکان، سال 1394

http://tbj.ssu.ac.ir/article-1-2146-fa.html

چکیده مقدمه: با دانستن میزان مصرف آب در بخش­های مختلف جامعه می­توان برنامه ریزی­های لازم در خصوص تأمین آب انجام داد. در مطالعه حاضر مقدار سرانه مصرف آب خانگی در خانوارهای شهر اردکان و رابطه آن با  متغیرهای مربوط به مصرف آب  بررسی گردید. روش بررسی: این مطالعه از نوع توصیفی- مقطعی بود و به روش نمونه­گیری خوشه­ای بر روی 300 خانوار شهر اردکان انجام گردید. پرسشنامه این تحقیق در برگیرنده اطلاعات زمینه­ای و همچنین متغیر­های مربوط به ساختمان بود. داده­های تحقیق با استفاده از آزمونهای Anova ,T-test  و ضریب همبستگی پیرسون، و با کمک نرم افزار16 spss مورد تجزیه و تحلیل قرار گرفت. یافته­ها: میانگین مصرف روزانه آب خانوارهای مورد مطالعه 32/518  لیتر و سرانه مصرف خانگی 16/147 لیتر محاسبه گردید. نتایج مطالعه نشان داد که میانگین مصرف آب خانوارها با متغیرهای بعد خانوار، سطح کل زمین، سطح زیربنا و درآمد کل خانوار ارتباط معناداری وجود دارد. نتیجه گیری: با توجه به بالا بودن سرانه مصرف آب خانگی در خانوارهای مورد مطالعه، ضروری است از طرق فرهنگ سازی، تبلیغات و اطلاع رسانی های کافی، نسبت به آگاه سازی مردم در خصوص عواقب ناشی از بحران کمبود آب اقدام و با ایجاد بستر مناسب آنها را ملزم  به اعمال اقدامات لازم(نظیر خرید کولر گازی)، تغییر عادات و رفتار نامناسب مصرف آب و صرفه جویی در مصرف آب نمود.

Title: بررسی تأثیر برنامه ریزی فرایند حسابرسی بر مدیریت کیفیت جامع

http://tbj.ssu.ac.ir/article-1-2626-fa.html

مقدمه: برنامه ریزی حسابرسی مبنای اساسی و مهم در اجرای فرایند حسابرسی است. هدف از پژوهش حاضر بررسی تأثیر برنامه ریزی حسابرسی بر مدیریت کیفیت جامع می باشد، که اجزای تشکیل دهنده برنامه‌ریزی حسابرسی شامل برنامه ریزی فرایند حسابرسی، تدوین طرح کلی حسابرسی و مستندسازی فرایند حسابرسی به عنوان متغیر مستقل بوده و مدیریت کیفیت جامع به عنوان متغیر وابسته می باشد. روش بررسی: داده ها بر اساس پرسشنامه دو بخشی که شامل برنامه ریزی فرایند حسابرسی تحت استاندارد شماره 300 و مدیریت کیفیت جامع به صورت طیف لیکرت پنج گزینه ای جمع آوری شدند. جامعه آماری این تحقیق بالغ بر 310 نفر از حسابرسان شاغل در مؤسسات حسابرسی عضو جامعه حسابداران رسمی ایران بوده که با استفاده از فرمول کوکران تعداد 172 نفر تعیین و به روش نمونه گیری تصادفی انتخاب شدند که در نهایت 111 پرسشنامه قابل استفاده جمع آوری گردید. یافته ها: این مطالعه نشان داد که برنامه ریزی فرایند حسابرسی بر مدیریت کیفیت جامع تاثیر معنادار (004/0=p) و مستقیم (358/0=r)، تدوین طرح کلی حسابرسی بر مدیریت کیفیت جامع تأثیر معنادار (001/0=p) و مستقیم (512/0=r) و مستندسازی فرایند حسابرسی بر مدیریت کیفیت جامع تأثیر معنادار (001/0=p) و مستقیم (557/0=r) دارد. نتیجه گیری: نتایج نشان می دهد که متغیر های برنامه ریزی حسابرسی، تدوین طرح کلی حسابرسی و مستندسازی فرایند حسابرسی بر مدیریت کیفیت جامع تأثیر آماری معناداری دارد،  به طوری که بیشترین تأثیر مربوط به متغیر مستندسازی فرایند حسابرسی بود.

Title: بررسی رابطه بین کیفیت خدمات و پاسخگویی بیمارستان و ارتباط ان با وفاداری بیماران بستری در بیمارستانهای دولتی شهر یزد درسال 1395

http://tbj.ssu.ac.ir/article-1-2260-fa.html

چکیده مقدمه: یکی از اولین و مهم ترین فاکتورها در بهبود مراقبت و فرآیندهای درمانی در مراکز ارائه دهنده خدمات اندازه گیری کیفیت خدمات و پاسخگویی از دید مشتریان می باشد. مقاله حاضر با هدف  بررسی رابطه بین کیفیت خدمات و پاسخگویی بیمارستان  و ارتباط ان با وفاداری بیماران در بیمارستانهای دولتی شهر یزد در سال 1395 انجام گردید. روش بررسی: مطالعه توصیفی-تحلیلی حاضر بر روی 200 نفر از بیماران مراجعه کننده به بیمارستان های دولتی شهر یزد در سال 1395 انجام گرفت. گردآوری اطلاعات با سه پرسشنامه کیفیت خدمات، پرسشنامه پاسخگویی WHO و پرسشنامه وفاداری انجام گرفت. تجزیه و تحلیل اطلاعات با استفاده از نرم افزار SPSS و به کمک آماره های توصیفی و آزمون آماری همبستگی پیرسون انجام شد. یافته ها: در بین ابعاد کیفیت خدمات بیشترین میانگین و انحراف معیار مربوط به بعد درک بیماری (0/67±3/95) و کمترین میانگین و انحراف معیار مربوط به بعد امور ناملموس (0/63±3/58) بود. در بین ابعاد پاسخگویی نیز بیشترین میانگین و انحراف معیار مربوط به بعد دسترسی به شبکه حمایت اجتماعی (0/88±2/26) و کمترین میانگین و انحراف معیار مربوط به بعد ارتباط با بیمار (0/84±2/55) بود. میانگین و انحراف معیار وفاداری نیز مقدار( 0/06±2/55)به دست آمد. همبستگی معنی داری بین کیفیت با پاسخگویی، کیفیت با وفاداری و وفاداری با پاسخگویی مشاهده شد(0/05>P). نتیجه گیری: نتایج نشان داد کیفیت و پاسخگویی بر وفاداری بیماران موثر بود. لذا مدیران باید ضمن برنامه ریزی در راستای ارتقای کیفیت خدمات، توجه به ابعاد ناملموس بیماران را افزایش و فرهنگ پاسخگویی را در بیمارستان ترویج دهند.

Title: بررسی تأثیر عدالت سازمانی ادراک‌شده بر بهروزی روان‌شناختی کارکنان جمعیت هلال‌احمر استان یزد

http://tbj.ssu.ac.ir/article-1-2500-fa.html

مقدمه: امروزه توجه سازمان‌های جهانی به بهداشت و بهروزی جامعه در بعد روانی بیشتر شده است، زیرا انسان‌ها در طول شبانه‌روز با مسائل جدیدی روبرو هستند که بهداشت روانشان تحت تأثیر سوء قرار می‌گیرد، ازاین‌رو توجه به این آسیب‌ها و عوامل پدیدآورنده‌ی سلامت روان باعث می‌شود تا در وهله‌ی اول بهداشت روانی هر فرد و درنهایت جامعه‌ای با سلامت روانی بالایی داشته باشیم. ازآنجایی‌که بخش اعظمی از شبانه‌روز افراد، در محیط کار می‌گذرد، لذا شناسایی تأثیر عوامل مختلف بر بهروزی روان‌شناختی در محیط کار بیش از فضاهای دیگر اهمیت پیدا می‌کند. هدف این پژوهش بررسی رابطه عدالت سازمانی ادراک‌شده و بهروزی روان‌شناختی هست. روش بررسی: این پژوهش با داشتن رویکرد علی، ازنظر هدف کاربردی و ماهیت گردآوری اطلاعات از نوع پیمایشی_ توصیفی است. برای سنجش متغیرهای مدل، پرسشنامه‌ای 48 سؤالی در نمونه‌ای 125 نفره از کارکنان جمعیت هلال‌احمر استان یزد مورداستفاده قرار گرفت. ضریب آلفای کرونباخ برای همه متغیرها در سطح قابل‌قبول قرار داشت و برای رسیدن به نتایج از روش مدل‌سازی معادلات ساختاری استفاده‌شده است. یافته‌ها: با توجه به مقدار ضریب تأثیر، عدالت سازمانی ادراک‌شده تأثیر مثبتِ معناداری بر بهروزی روان‌شناختی کارکنان دارد و تأثیر مثبت ابعاد عدالت توزیعی و بین فردی بر بهروزی روان‌شناختی نیز تائید شد. نتیجه‌گیری: مدیران سازمان‌ها می‌توانند با ایجاد زمینه‌های درک عدالت برای کارکنان خود در ابعاد مختلف توزیعی، رویه‌ای، اطلاعاتی و بین فردی زمینه‌ساز شرایطی شوند که کارکنان، ضمن احساس خرسندی و شادابی، با تأکید بر نکات مثبت بهداشت فیزیکی و روانی خود در جهت بالفعل کردن استعدادهایشان تلاش کنند.

Title: بررسی تاثیر آموزش بر اساس تئوری رفتار برنامه ریزی شده بر مصرف میوه و سبزیجات در زنان روستایی چابهار

http://tbj.ssu.ac.ir/article-1-1760-fa.html

مقدمه: شواهد متعددی  وجود دارد که نشان می دهد مصرف میوه و سبزیجات نقش محافظتی در مقابل بیماری های عمده بازی می کند. این مطالعه با هدف بررسی تاثیر آموزش با استفاده از تئوری رفتار برنامه ریزی شده برمصرف میوه و سبزیجات در  زنان روستایی چابهار انجام شد. روش بررسی: این تحقیق یک مطالعه تجربی مورد  شاهدی بود. 152 نفر از زنان به روش نمونه گیری چند مرحله ای از بین روستاهای بخش مرکزی چابهار انتخاب شدند.داده ها در دو مقطع زمانی قبل و بعد از آموزش از طریق پرسشنامه جمع آوری شد. آموزش شامل یک جلسه بحث گروهی ، یک جلسه سخنرانی، یک کتابچه آموزشی و یک پوستر آموزشی توسط رابطین برای گروه مداخله اجرا شد.داده های توسط نرم افزار  spssنسخه 19 مورد تجزیه و تحلیل قرار گرفت. یافته ها: میانگین سنی زنان شرکت کننده در این مطالعه 66/26 با انحراف معیار72/6 بود. اختلاف معنی داری از لحاظ ویژگی های دموگرافیک در دو گروه مشاهده نگردید. در گروه مداخله تغییرات معنی داری در تمام  متغیرهای مورد مطالعه ایجاد شد در حالیکه این تغییرات در گروه کنترل معنی دار نبود. تغییر ساختارهای نگرش وقصد رفتاری مجموعا 12 % رفتار و هنجارهای انتزاعی ،آگاهی و کنترل رفتاری درک شده باهم 25 % از قصد مصرف میوه و سبزیجات را پیش بینی کردند. نتیجه گیری: مداخلات آموزشی طراحی شده بر اساس تئوری رفتار برنامه ریزی شده در این مطالعه باعث ارتقای قصد و رفتار مصرف میوه و سبزیجات در زنان روستاهای چابهارگردید. اما میزان تغییر  رفتار کمتر از تغییر قصد رفتاری بود. این موضوع بار دیگر نشان می دهد که هر قصدی نمی تواند منجر به رفتار شود و بایستی در راستای تغییر رفتار موانع رفتاری نیز مورد توجه قرار گیرد و در مداخلات آموزشی حتما اقداماتی در این راستا صورت گیرد.
